# The Increase of Triterpene Saponin Production Induced by *Trans*-Anethole in Hairy Root Cultures of *Panax quinquefolium*

**DOI:** 10.3390/molecules23102674

**Published:** 2018-10-17

**Authors:** Ewa Kochan, Piotr Szymczyk, Łukasz Kuźma, Grażyna Szymańska, Anna Wajs-Bonikowska, Radosław Bonikowski, Monika Sienkiewicz

**Affiliations:** 1 Pharmaceutical Biotechnology Department, Medical University of Lodz, Muszyńskiego 1, 90-151 Lodz, Poland; piotr.szymczyk@umed.lodz.pl (P.S.); grazyna.szymanska@umed.lodz.pl (G.S.); 2 Department of Biology and Pharmaceutical Botany, Medical University of Lodz, Muszyńskiego l, 90-151 Lodz, Poland; lukasz.kuzma@umed.lodz.pl; 3 Institute of General Food Chemistry, Biotechnology and Food Science, Lodz University of Technology, Stefanowskiego St. 4/10, 90-924 Lodz, Poland; anna.wajs-bonikowska@p.lodz.pl (A.W.-B.); radoslaw.bonikowski@p.lodz.pl (R.B.); 4 Department of Allergology and Respiratory Rehabilitation, Medical University of Lodz, 90-151 Lodz, Poland; monika.sienkiewicz@umed.lodz.pl

**Keywords:** hairy root cultures, *trans*-anethole, elicitation

## Abstract

In vitro cultivation is an effective way to increase pharmaceutical production. To increase ginsenoside production in hairy root cultures of American ginseng, the present study uses *trans*-anethole as an elicitor. The content of nine triterpene saponins was determined: Rb1, Rb2, Rb3, Rc, Rd, Rg1, Rg2, Re and Rf. *Trans*-anethole was found to stimulate saponin synthesis regardless of exposure time (24 and 72 h). Twenty-four hour exposure to 1 μmol *trans*-anethole in the culture medium resulted in the highest increase of total saponin content (twice that of untreated roots), and optimum accumulation of Rb-group saponins, with ginsenoside Rc dominating (8.45 mg g^−1^ d.w.). In contrast, the highest mean content of protopanaxatriol derivatives was obtained for 10 μmol *trans*-anethole. The Re metabolite predominated, reaching a concentration of 5.72 mg g^−1^ d.w.: a 3.9-fold increase over untreated roots. Elicitation with use of *trans*-anethole can therefore be an effective method of increasing ginsenoside production in shake flasks.

## 1. Introduction

Ginsenosides, triterpene saponins, are produced as secondary plant metabolites in the genus *Panax.* Generally, they are classified as protopanaxadiol (PPD) derivatives (Rb1, Rh2, Rb3, Rc, Rd, 20(S)-Rg3, Rb2) and protopanaxatriol (PPT) derivatives (Re, Rf, Rg1, Rg2, Rg3, Rh1) according to their aglycone structures. PDD-group saponins have glycosidic bonds at the C-3 and/or C-20 hydroxyl groups and linear linkage of glucosyl chains, while the PPT-group saponins have common glycosidic bonds at C-6 and/or C-20 at a maximum of two glycosyl chains [1]. The hydroxyl groups can be free, or they can be bound to monomeric, dimeric, or trimeric sugars. Ginsenosides are considered to be the most pharmacologically active compounds produced by ginseng species and are known to have regulatory effects on the nervous, endocrine, cardiac, and immune systems. In addition, ginsenosides possesses vasorelaxation, antioxidant, anti-inflammation, anticancer, radioprotective and antiaging properties [2,3,4]. 

Currently, these metabolites are obtained from field crops on an industrial scale. However, ginseng is a forest dweller, and for its successful cultivation, it should be protected against direct sunlight and from strong winds, and the soil should be fertile, rich in nutrients, airy with a permeable substrate and covered with mulch. Moreover, the cultivation of the widest-known ginseng species, such as *P. ginseng*, *P. quinquefolium* or *P. notoginseng*, requires a minimum of three years before harvest. All these conditions make ginseng cultivation difficult and costly. Unfortunately, chemical synthesis of ginsenosides is also inefficient and expensive [5]. 

To address these problems, research is being performed in obtaining ginsenosides from in vitro plant cultures such as transgenic roots. Our own previous studies demonstrate that hairy root cultures of *P. quinquefolium* can produce comparable amounts of ginseng saponins to naturally grown roots, but in a significantly shorter time, with only 28 days being needed for cultivation. This increased production was made possible using an elicitation protocol [6,7]. Elicitation is regarded as one of most effective biotechnological tools for intensifying the biosynthesis of useable plant metabolites, with fungal cell extracts, polysaccharides from fungal and plant cells, jasmonates and other hormones, UV radiation, hyperosmotic stress, temperature, and heavy metal salts being commonly used to improve the yield secondary metabolites in a range of hairy root cultures [8,9,10]. New natural compounds that could play elicitor, are still being sought. An important criterion for the selection of such compounds is documented their biological activity and lack of toxicity. *Trans*-anetol, not used as an elicitor, was chosen for our research.

The phenylpropene derivative *trans*-anethole occurs widely in nature in essential oils. It is produced in large quantities as a secondary metabolite of plants of the *Apiaceae* family, such as anise and fennel, as well as by anise myrtle (*Myrtaceae*) and by star anise (*Illiciaceae*), and in various amounts by many other plants. Due to presence of high levels of *trans*-anethole, the essential oils obtained from fennel and anise or star anise possesses antibacterial and antifungal activity and are used in the food industry as a measure to prevent spoilage [11,12]. Microbiological studies on fennel essential oil containing mainly *trans*-anetole (77.9%) found it to increase the inhibition zone around cefoxitin, mupirocin, co-trimoxazole and ciprofloxacin against *Staphylococcus aureus* isolated from carriers [13]. *Trans*-anethole has been used in natural medicine for a long time due to its range of valuable biological activities, including its anithelminic, anti-inflammatory and gastroprotective properties [14,15,16]. *Trans*-anethole is regarded as a safe active compound: its LD_50_ administered orally in mice, rats and guinea pigs was found to be between 2090 mg/kg and 3050 mg/kg [17].

While our previous studies have used typical elicitors such as yeast extract or methyl jasmonate [6,7], the aim of the present study was to determine whether *trans*-anethole can be used as potential new elicitor to enhance triterpene saponin production in *P. quinquefolium* hairy roots cultivated in shake flasks. Such use of essential oil components for elicitation is currently not documented. However, as the yield of the secondary metabolite is influenced not only by the type and dose of the elicitor, but also by the treatment schedule [18], our study also examines the optimum elicitation time of *trans*-anethole for effective ginsenoside biosynthesis in the studied cultures.

## 2. Results

This study examines the effect of 24-h and 72-h elicitation by *trans*-anethole (*t-*A) on triterpene saponin content in *Panax quinquefolium* hairy roots cultured in shake flasks. After elicitation, the following ginsenosides were measured: Rb1, Rb2, Rb3, Rc, Rd (protopnaxadiol derivatives), and Rg1, Re, Rg3, Rf (protopanaxatriol derivatives).

**Figure 1:**
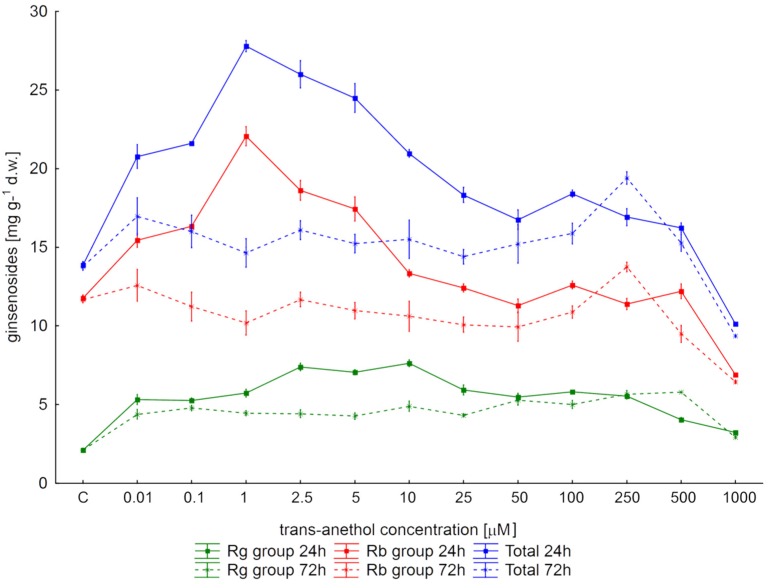
The effect of *trans*-anethole concentration on ginsenoside level in *Panax quinquefolium* hairy roots after 24- and 72-h elicitation. Rb-group protopanaxadiol derivatives, Rg group- protopanaxatriol derivatives.

The highest total content of all tested saponins (27.79 mg g^−1^ d.w.) was observed after 24 h of elicitation with 1 µM *trans*-anethole (Figure 1), where the ginsenoside level was twice that observed in the control samples. Elevated saponin levels were also observed following treatment with the other concentrations of *trans-*anethole, except 1000 µM. Prolonging the elicitation time to 72 h resulted in a lower yield of ginsenoside biosynthesis independent of *t-*A concentration. The maximum level of studied metabolites (19.4 mg g^−1^ d.w.) was 30% lower than that obtained after 24 h and required a 250-times higher *trans-*anethole concentration. 

Similar trends in triterpene saponin content were obtained for the Rb-group ginsenosides, expressed as the sum of Rb1, Rc, Rb2, Rb3 and Rd (Figure 1). Greater amounts of protopanaxadiol derivatives were observed following the shorter elicitation time. In addition, their yield also responded to elicitor concentration to in a similar way as did the total examined metabolite content: their level initially increased with elicitor concentration, achieving the highest production (22.07 mg g^−1^ d.w.) at 1 µM *trans*-anethole, with further increases in elicitor concentration resulting in a gradual decrease in yield. 

A different relationship was observed for protopanaxatriol derivatives (expressed as sum of Re and Rg1). Efficient Rg group saponin accumulation was found, with 6.99–7.62 mg g^−1^ d.w. ginsenosides being seen following 24-h elicitation with 2.5–10 µM *trans*-anethole in the medium. This level of saponin was 3.3–3.59 times higher than in untreated controls (Figure 1).

Obtained results indicated that the yields of the Rb-group ginsenosides exceeded those of the Rg group (Rb-group/ Rg group > 1) regardless of the elicitor concentration and its exposure time. 

**Figure 2:**
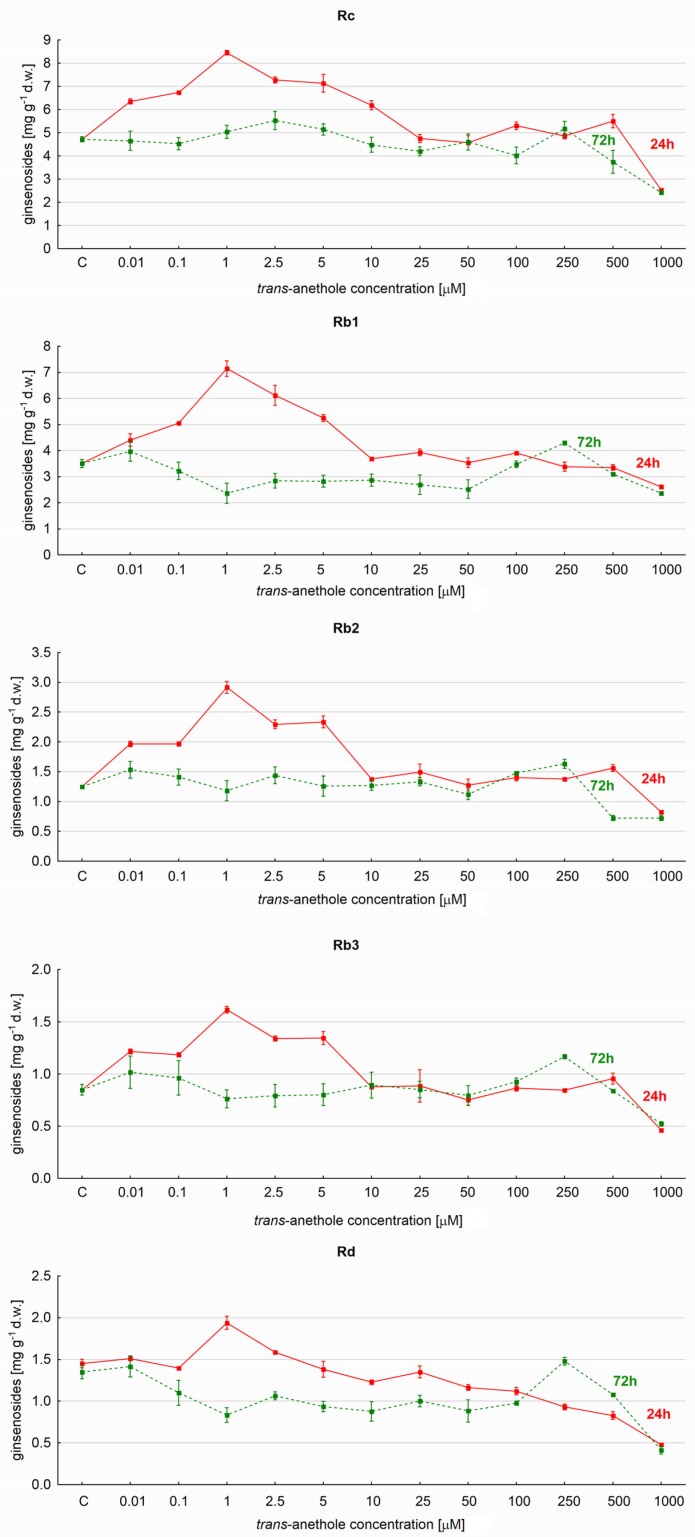
The effect of *trans*-anethole concentration on the production of Rc, Rb1, Rb2, Rb3 and Rd ginsenosides in *Panax quinquefolium* hairy roots after 24- and 72-h elicitation.

The levels of nine individual ginsenosides were also determined, including Rc, Rb1, Rb2, R3, Rd, Rg1 and Re. It was found that the 24-h elicitation period induced significantly greater production of individual triterpene saponins than the longer exposure time (Figure 2), with an elicitor concentration of 1 µM resulting in the greatest percentage increases relative to controls: Rc (80.6%), Rb1 (98.3%), Rb2 (137.1%), Rb3 (97.6%) and Rd levels (33.79%). However, exposure to increasing *trans*-anethole concentrations greater than 1 µM caused a gradual reduction in yield to control levels or below. Interestingly, each compound was found to have a different threshold concentration of *t*–A above which the saponin amount was depressed below control values, for example 5 µM for Rd, 250 µM for Rb1, and for the remaining 1000 µM (Figure 2).

Ginsenoside Rc demonstrated higher yields than the other tested compounds, with the greatest yield being 8.54 mg g^−1^ d.w., accounting for nearly 30% of all saponin content. A similar level was determined for metabolite Rb1 (7.14 mg g^−1^ d.w., 25.7% of all studied compounds). Of the remaining ginsenosides of the Rb-group, the yields were 2.94 (Rb2), 1.62 (Rb3) and 1.94 mg g^−1^ d.w. (Rd).

**Figure 3:**
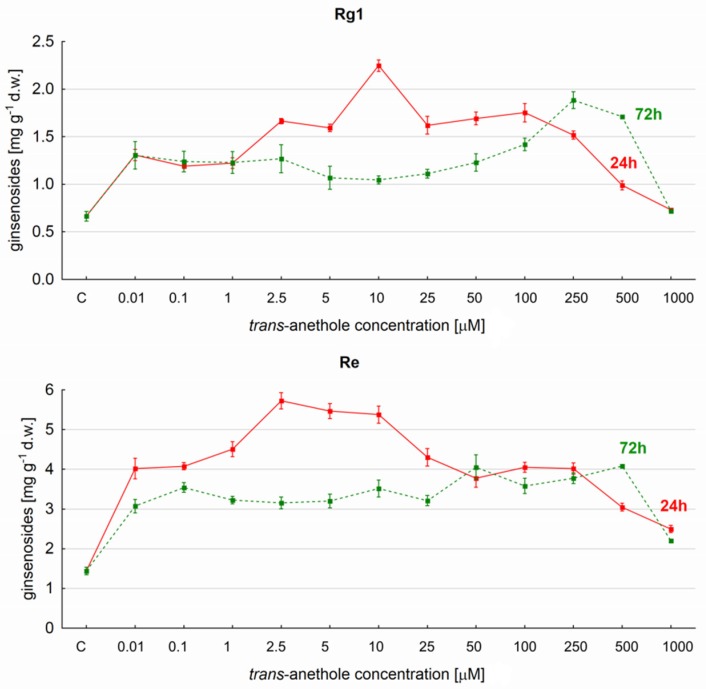
The effect of *trans*-anethole concentration on the production of the Re and Rg1 ginsenosides in *Panax quinquefolium* hairy roots after 24- and 72-h elicitation.

As with all protopanaxadiol derivatives, saponins Re and Rg1 demonstrated greater yields following 24-h elicitation than 72 h. However, Re production was found to be stable in the range 5 to 10 µM *trans*-anethole (Figure 3), with Re content remaining between 5.37 and 5.72 mg g^−1^ d.w. between these concentrations. This yield was around four times greater than the control sample values.

The second most abundant compound observed following elicitation by 10 µM *trans*-anethole, following Re, was Rg1. Following elicitation, Rg1 levels reached 2.25 mg g^−1^ d.w. and accounted for 246% of control values. 

**Figure 4:**
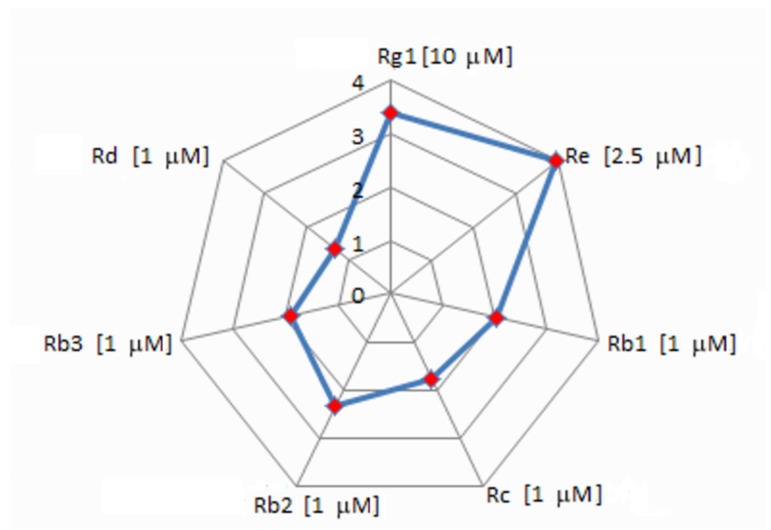
The maximum increase of ginsenosides in relation to untreated samples, after *t*-A elicitation.

It was found that *trans*-anethole stimulates the synthesis of the studied triterpene saponins. Their level was higher than control values for most used *trans*-anethole concentrations. The maximum increase of ginsenosides in relation to untreated samples, after *t*-A elicitation, demonstrates Figure 4.

## 3. Discussion

The use of essential oils or their individual components to enhance the production of secondary metabolites in plant in vitro cultures remains an unexplored area in research. The present study represents the first examination of the use of *trans*-anethole as an elicitor of triterpene saponin production in *P. quinquefolium* hairy root cultures. Its purpose was to determine the influence of different concentrations and exposure times of *trans*-anethole on the resulting ginsenoside content in studied cultures.

It was found that even in small concentrations, *trans*-anethole can be used as an elicitor to improve triterpene saponin production. The optimal *trans-*anethole concentration for efficient biosynthesis of sum of seven studied ginsenosides in the hairy root cultures was found to be 1 µM. The total ginsenoside level was 27.79 mg g^−1^ d.w. following elicitation, and this represented an increase in production of almost 100% compared to controls. A similar total content of studied ginsenosides (Rb1 + Rb2 + Rc + Rd + Re + Rg1) i.e., 27.23 mg g^−1^ d.w. was noted after using methyl jasmonate (MJ), one of the most commonly used and most effective elicitors, at 250 µM [7]. Kim et al. [19] indicate that 100 µM of MJ may be optimal for ginsenoside accumulation in *P. ginseng* adventitious root. In addition, 500 and 200 µM MJ were found to be most suitable concentrations for obtaining high yields of ginseng saponins in *P. ginseng* and *P. notoginseng* suspension cultures, respectively [20,21]

Our present findings demonstrate that significantly lower concentrations of *trans-*anethole than MJ are enough to improve ginsenoside production. Similar observations have been found in relation to yeast extract used as elicitor for *P. quinquefolium* hairy root cultures [6].

Various elicitors appear to influence the accumulation of secondary compounds in different ways, as indicated by the levels of individual ginsenosides in *P. quinquefolium* transformed root cultures when the elicitors are applied at optimal concentrations. Rc and Rb1 saponins predominated following *trans-*anethole application, while Rb1 and Re were at the highest levels following MJ or yeast extract stimulation [6,7]. In addition, treatment with 1 µM *trans*-anethole resulted in Rc and Rb1 levels twice those of controls, and Rg1 and Re levels three times higher. MJ treatment resulted in an approximately five-fold increase of some ginsenosides in hairy and adventitious root cultures of *P. ginseng* [19,22,23] and a nine-fold increase of Rb1 in *P. notoginseng* suspension cell cultures [24]. Other elicitors such as chitosan, vanadyl sulfate or Tween 80 also stimulated the accumulation of individual ginsenosides in *P. ginseng* hairy root cultures [22,25]. Hence, ginseng saponins can be stimulated to different degrees depending on elicitor type. In addition, the dose of the elicitor strongly influences the intensity of ginsenoside production and should be determined empirically. 

Besides elicitor specificity and concentration, time of elicitation is also a very important factor in determining the synthesis of secondary metabolites. The investigation described in this paper indicated that 24-h elicitation results in better ginsenoside accumulation than the 72-h exposure time. In contrast, significantly longer elicitation times were required for MJ (seven days) and yeast extract (three days) to effectively produce triterpene saponins in the same cultures [6,7]. Meanwhile Palazon et al. [22] reported that 3-,13-or 28-day elicitation of vanadyl sulfate enhanced production of Rb-group ginsenosides in *P. ginseng* hairy root.

Our findings suggest that *trans*-anethole can be used as an elicitor. It is widely believed that elicitors act by mobilizing the biosynthesis of secondary metabolites (Figure S1) [26]. This process usually begins at the plasma membrane or endomembrane, where specific receptors recognizable by the elicitor are present. The elicitor first binds to a suitable receptor; following this, a signal is recognized, and a response is generated. The molecule of the elicitor is identified as a pathogen thus activating plant resistance and pathogen avirulence genes. The products of these genes play a key role in this step [27]. 

Following this, second messengers are created that amplify the signal for other downstream reactions. The successive changes induced by the elicitor proceed as Figure S1: reversible phosphorylation and dephosphorylation of cell membrane proteins and cytosolic proteins, cytosolic [Ca^+^] burst; alkalization of the extracellular environment and acidification of the cytoplasm, caused by Cl^−^ and K^+^ efflux/H^+^ influx; MAPK (mitogen-activated protein kinase) and NADPH oxidase activation, production of reactive oxygen and nitrogen molecules (ROS and RNS); early expression of defense genes; production of jasmonates; the expression of genes invoking enzymatic reactions that in turn reprogram metabolic pathways and lead to secondary metabolite accumulation [9,26]. It is highly probable that *trans*-anethole acts according to the above mechanism; however, further molecular investigations are necessary.

## 4. Materials and Methods

### 4.1. Hairy Root Culture

The Panax quinquefolium hairy root cultures were obtained by Agrobacterium rhizogenes ATCC 15834 transformation as previously described [28]. The cultures grew in shake Erlenmeyer flasks with 80 mL of modified B-5 medium [29,30] with 30 g L^−1^ sucrose but without hormone. They were placed on rotary shakers (100 rpm) at 26 °C ± 2 °C in darkness. The mean inoculum size was about 310 mg fresh weight (f.w.) and 29.1 mg dry weight (d.w.).

### 4.2. Elicitation Process

In this experiment *trans-*anethole in concentrations of: 0, 0,01; 0,1; 1; 2,5; 5; 10; 25; 50, 100, 250, 500, 1000 µM was applied as an elicitor. The compound was added to medium in the stationary phase, on the 28th day of culture. After 24 and 72 h of elicitation, hairy roots were harvested and ginsenoside content was determined. 

*Trans-*anethole of 99% purity was obtained from Sigma-Aldrich, Germany. 

### 4.3. Ginsenoside Content Determination

#### 4.3.1. Sample Preparation

Roots dried at room temperature were used for ginsenoside extraction. Crude methanolic extracts of ginsenosides were purified using an SPE (Solid Phase Extraction) column as described previously [6]. 

#### 4.3.2. Standard Solution

The mixture of ginsenosides (Rb1, Rb2, Rb3, Rc, Rd, Re, Rg1, Rg2, Rf) was purchased from Aldrich Sigma (Darmstadt, Germany). The concentration of each metabolite was 100 µg mL^−1^. The standard curves were obtained by applying different volumes of ginsenoside mixtures in the HPLC apparatus. The peak area of each ginsenoside standard was recorded. The standard curve was calculated according to the peak area and concentration. In addition, the LOD (limit of detection) and LOQ (limit of the detailed regression equation and validation data) of each standard were recorded (Table 1).

#### 4.3.3. HPLC analysis of ginsenosides

The extracts were studied by HPLC. The ginsenosides were dissolved in 1 mL of HPLC grade methanol (J.T. Baker, Deventer, The Netherlands), filtered through 0.2 μm pore diameter Milipore^®^-FG Hydrophobic Fluoropore filters (PTFE) (Merck, Darmstadt, Germany)) and applied to an Agilent Technology 1200 (Agilent Technologies Inc., Santa Clara, CA, USA) liquid chromatography apparatus. It was equipped with a ZORBAX Eclipse XDB-C18 column (150 × 4.6 mm, 5 μm), Quat Pump, UV-VIS DAD type detector and autosampler. The Agilent Technology apparatus was combined with Agilent ChemStation 2001–2010 software (Agilent Technologies Inc., Santa Clara, CA, USA). A two-component mobile phase composed of acetonitrile (A) (J.T. Baker, Deventer, The Netherlands) and water (B) (J.T. Baker, Deventer, The Netherlands) was used for determination of ginsenosides. The following gradient elution program was applied: 0–16 min: 18% A, 82% B; 16–28 min: 30% A, 70% B; 28–60 min: 32% A, 68% B; 60–64 min: 80% A, 20% B; 64–68 min: 18% A, 72% A. The flow rate was 2 mL min^−1^. Ginsenoside detection was performed at a wavelength of 203 nm. The quantitative content of ginsenosides (mg g^−1^ d.w.) was determined by comparing retention time and peak areas between standards and samples. 

### 4.4. Statistical Analysis

All treatments were performed in triplicate. Data was analyzed using the Kruskal-Wallis test. Any relationships were considered significant at *p* ≤ 0.05. Statistica Version 13.1 software was used for all statistical analyses (STATSoft, Tulsa, OK, USA).

## 5. Conclusions

This study evaluates the effect of different concentrations of *trans*-anethole on the production of ginsenosides in transformed root cultures of *Panax quinquefolium* in shake flasks. It also examines the effect of changing elicitor exposure time. *Trans*-anethole can be used as elicitor to increase triterpene saponin production in the studied cultures. 

The results show that ginsenoside synthesis was enhanced to a greater degree by elicitation for 24 h than 72 h. 

The optimal concentration of trans-anethole needed for efficient biosynthesis of the combined studied ginsenosides was found to be 1 µM: At this concentration, total saponin content was twice that observed in the untreated samples, and optimal synthesis of individual saponins from the Rb-group was also observed. The optimal concentration for the Re ginsenosides was found to be in the range of 2.50–10 µM while Rg1 production was optimized at 10 µM. The most plentiful saponins were Rc and Rb1.

## Figures and Tables

**Table 1:** The ginsenoside standard curves.

Ginsenosides	Regression Equation	r^2^	LOD (µg mL^−1^)	LOQ (µg mL^−1^)
Rb1	y = 206.23x − 0.83	0.9992	2.02	6.66
Rb2	y = 149.39x − 0.19	0.9988	1.78	5.87
Rb3	y = 699.95x − 0.55	0.9988	0.26	0.86
Rc	y = 142.57x − 0.40	0.9981	1.35	4.46
Rd	y = 166.08x − 0.46	0.9993	1.80	5.94
Rg1	y = 181.38x + 0.19	0.9989	0.55	1.82
Rg2	y = 181.18x + 0.32	0.9989	0.29	0.96
Re	y = 155.38x − 0.21	0.9989	1.44	4.75
Rf	y = 202.63x − 0.98	0.9998	0.18	0.59

## Supplementary Materials

The following are available online, Figure S1: Schematic representation of the possible responses of cells to elicitation. R: receptor; PL: phospholipase; MAPKs: mitogen activated protein kinases; ROS: reactive oxygen species; RNS: reactive nitrogen species; TF: transcription factors [according to 9].
