# Supplementary material for: The Increase of Triterpene Saponin Production Induced by Trans-Anethole in Hairy Root Cultures of Panax quinquefolium
Source: Molecules. 2018 Oct 17;23(10):2674. doi: 10.3390/molecules23102674 (PMC6222429; doi:10.3390/molecules23102674)
Supplement: Supplementary file 1 [file molecules-23-02674-s001.pdf]

Article

The increase of triterpene saponin production induced by trans-anethole in hairy root cultures of *Panax quinquefolium*

Ewa Kochan [1,*], Piotr Szymczyk [1], Łukasz Kuźma [2], Grażyna Szymańska [1], Anna Wajs-Bonikowska [3], Radosław Bonikowski [3], Monika Sienkiewicz [4]

[1] Pharmaceutical Biotechnology Department, Medical University of Lodz, Muszyńskiego 1, Lodz 90-151, Poland; ewa.kochan@umed.lodz.pl (E.K.); piotr.szymczyk@umed.lodz.pl (P.S.); grazyna.szymanska@umed.lodz.pl (G.S.)

[2] Department of Biology and Pharmaceutical Botany, Medical University of Lodz, Muszyńskiego l, Lodz 90-151, Poland; lukasz.kuzma@umed.lodz.pl

[3] Institute of General Food Chemistry, Biotechnology and Food Science, Lodz University of Technology, Stefanowskiego St. 4/10, 90-924 Lodz, Poland; anna.wajs-bonikowska@p.lodz.pl (A.W.-B.); radoslaw.bonikowski@p.lodz.pl (R.B.)

[4] Department of Allergology and Respiratory Rehabilitation, 2nd Chair of Otolaryngology Medical University of Lodz, Poland; monika.sienkiewicz@umed.lodz.pl

* correspondence: ewa.kochan@umed.lodz.pl

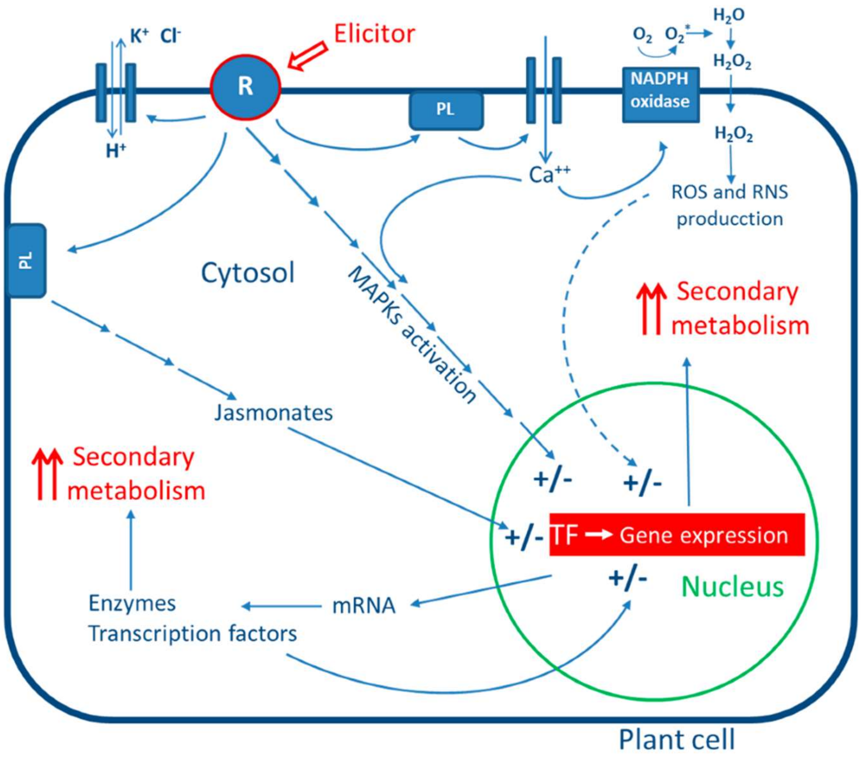

Figure S1. Schematic representation of the possible responses of cells to elicitation. R: receptor; PL: phospholipase; MAPKs: mitogen activated protein kinases; ROS: reactive oxygen species; RNS: reactive nitrogen species; TF: transcription factors [according to 9].
